# Two major quantitative trait loci control wheat dwarf virus resistance in four related winter wheat populations

**DOI:** 10.1007/s00122-023-04349-3

**Published:** 2023-04-07

**Authors:** Maria Buerstmayr, Hermann Buerstmayr

**Affiliations:** Institute of Biotechnology in Plant Production, University of Natural Resources and Life Sciences, Vienna, Konrad Lorenz Straße 20, 3430 Tulln, Austria

## Abstract

**Key message:**

*Qwdv.ifa-6A* on chromosomes 6AL and *Qwdv.ifa-1B* on chromosome 1B are highly effective against wheat dwarf virus and act additively when combined.

**Abstract:**

Wheat dwarf virus (WDV) is among the most damaging viral pathogens. Its prevalence has increased substantially in recent years, and it is predicted to increase even further due to global warming. There are limited options to control the virus. Growing resistant cultivars would safeguard crops, but most current wheat cultivars are highly susceptible. Thus, the aim of this study was to dissect the genetic architecture of WDV resistance in resistant germplasm and to identify quantitative trait loci (QTL) to support resistance breeding. QTL mapping was conducted using four related populations comprising 168, 105, 99 and 130 recombinant inbred lines. Populations were evaluated under field conditions for three years. Natural infestation was provoked by early autumn sowing. WDV symptom severity was visually assessed at two time points in spring. QTL analysis revealed two highly significant QTL with the major QTL *Qwdv.ifa-6A* mapping to the long arm of chromosome 6A between markers Tdurum_contig75700_411 (601,412,152 bp) and AX-95197581 (605,868,853 bp). *Qwdv.ifa-6A* descends from the Dutch experimental line SVP-72017 and was of high effect in all populations, explaining up to 73.9% of the phenotypic variance. The second QTL, *Qwdv.ifa-1B*, mapped to chromosome 1B and is putatively associated with the 1RS.1BL translocation, which was contributed by the CIMMYT line CM-82036. *Qwdv.ifa-1B* explained up to 15.8% of the phenotypic variance. *Qwdv.ifa-6A* and *Qwdv.ifa-1B* are among the first identified highly effective resistance QTL and represent valuable resources for improving WDV resistance in wheat.

**Supplementary Information:**

The online version contains supplementary material available at 10.1007/s00122-023-04349-3.

## Introduction

Wheat dwarf virus (WDV) disease was first documented in Europe in the former Czechoslovakia during the 1960s (Vacke 1961) but may have been the cause of severe damage in wheat in Sweden as early as 1912 (Lindsten and Lindsten 1999). After the 1960s wheat dwarf (WD) became a problematic disease in many European countries (Mishchenko et al. 2022; Schubert et al. 2014; Trzmiel 2020) and was also reported in the Middle-East (Köklü et al. 2007), Iran (Behjatnia et al. 2011; Parizipour et al. 2017), Africa (Kapooria and Ndunguru 2004), Western-Asia (Ekzayez et al. 2011) and Asia (Xie et al. 2007). Infection with WDV manifests in dwarfism of varying degrees, reduced winter hardiness, streaky to blotchy lightening or yellowing of leaves and reduced or no heading. WDV may even lead to premature plant death and drastic yield losses (Lindblad and Waern 2002; Lindsten and Lindsten 1999; Vacke 1961).

WDV is a DNA virus species of the genus *Mastrevirus* belonging to the family Geminiviridae (Fauquet and Stanley 2003). The virus uses the leafhopper *Psammotettix alienus* (Homoptera: *Cicadellidae*) as its vector (Lindsten and Lindsten 1999; Vacke 1961). WDV is transmitted to its host in a persistent-circulative non-propagative manner (Lindblad and Sigvald 2004) meaning that the virus has a short, latent period, and the leafhoppers remain infectious throughout their lifetime, the virus does not multiply within the insect and cannot be passed on to offspring (Nault 1997). Hence, new populations of virus-bearing leafhoppers will be generated every spring by nymphs feeding on infected host plants. WDV has a wide range of monocotyledonous hosts, including economically important cereals such as wheat, barley, oat, rye and triticale and some wild grasses (Vacke 1972). The main virus sources are infected field stands and self-sown ‘volunteer’ cereal plants in lay fields (Manurung et al. 2004; Mehner et al. 2003), while infected wild grasses are less important but may act as long-term reservoir for the virus (Ramsell et al. 2008; Yazdkhasti et al. 2021). Primary infection of winter wheat occurs in autumn when infected adult leafhoppers transfer the virus into newly sown crops. Secondary infection occurs in spring and early summer via newly hatched nymphs that absorb and spread the virus from WDV infested plants (Lindblad and Sigvald 2004; Lindblad and Waern 2002). Plants are most susceptible to WDV when infection occurs during the one to three leave stage (Vacke 1972), while milder symptoms occur when infection occurs at later development stages. Wheat plants become resistant when first nodes are detectable (Lindblad and Sigvald 2004; Lindblad and Waern 2002; Lindsten and Lindsten 1999). Leaf hoppers are highly mobile provided daily maximum temperatures exceed 15 °C, whereas their activity slows down and finally stops at temperatures below 10 °C (Lindblad and Sigvald 2004). Warm weather during autumn results in both, higher density of WD diseased plants and greater probability for eggs to overwinter on virus contaminated leaves (Lindblad and Arenö 2002). Consequenctly, winter wheat from fields sown in early autumn suffers more from WDV than wheat from fields sown in late autumn (Lindblad and Waern 2002). It is predicted that the prevalence of viral diseases will increase, and their control will become more difficult due to climatic instability resulting from global warming (Jones 2021; Roos et al. 2011; Trebicki 2020). Global warming together with new cultivation practices, such as early sowing, reduced tillage, and incorporation of fallow in the crop rotation can have strong effects on leaf hopper populations and WDV epidemiology, thereby increasing the risk of severe disease outbreaks with yield losses up to 90% (Lindblad and Sigvald 2004; Lindblad and Waern 2002; Lindsten and Lindsten 1999).

Currently, no approved insecticide against *P. alienus* is available in the European Union (Pfrieme et al. 2022). Moreover, effectiveness of chemical control measures would be limited due to the high mobility of the leaf hoppers. Removing plant residues, ploughing after harvesting, elimination of volunteer cereals and late sowing of winter cereals or early sowing of spring cereals are currently the only agronomic measures for controlling this disease. However, late sowing in autumn may not be an option when the bunts are a problem, since late sowing increases infectivity of the common bunt (*Tilletia caries* and *T. laevis*) and dwarf bunt (*T. controversa*) pathogens, as germinating seedlings are particularly receptive to *Tilletia* spp. infection under cool temperature conditions (Goates 1996). Genetic resistance would be the preferred cost-efficient and environmentally friendly alternative, but most current wheat cultivars are susceptible or highly susceptible to WDV (Pfrieme et al. 2022; Sirlova et al. 2005; Vacke and Cibulka 2000). Nonetheless, there is variation in the wild and domesticated wheat gene pool (Nygren et al. 2015; Pfrieme et al. 2022) and to date the Hungarian cultivars ‘Mv Vekni’ and ‘Mv Dalma’ (Benkovics et al. 2010), genebank accession ‘PI 245511’ and the Russian winter wheat cultivar ‘Fisht’ showed moderate to high partial resistance to WDV (Pfrieme et al. 2022). Very little is known about the genetic control of WDV. The only QTL analysis published so far was Pfrieme et al. (2022) who conducted a genome-wide association study (GWAS) using 250 winter wheat accessions and identified 35 putative QTL, of which 14 were confirmed in bi-parental populations, suggesting quantitative regulation of resistance to WDV.

By monitoring early and late sown winter wheat trials in our Fusarium head blight (FHB) nurseries we repeatedly observed that some of the early sown wheat lines developed severe WDV symptoms, whereas a few lines remained almost unaffected. From this material, two lines with repeatedly low WDV severity were selected and crossed with susceptible winter wheat cultivars/lines to develop four related mapping populations. Using early sowing and relying on natural infections, we evaluated these populations for WDV severity in three consecutive years allowing us to discover, validate and compare efficacy of the identified WDV resistance QTL.

## Materials and methods

### Plant materials

Recombinant inbred line (RIL) populations descending from the reciprocal crosses Capo/SVP-72017 and SVP-72017/Capo were initially used to study the inheritance of resistance to FHB (Buerstmayr et al. 2000). As a side effect, constant and clear variation in WDV severity was observed between lines within these populations in early autumn-sown field experiments, whereas the same materials remained unaffected in later sown field trials. Among these, line A39 (Capo/SVP-72017) and A40 (SVP-72017/Capo) was selected for their low expression of WDV symptoms and used as resistance donors in the following four bi-parental populations Midas/A40 (abbreviated to MI/A40), Mulan/A40 (abbreviated to MU/A40), P1314/A40, and A39/P1314. Single seed descents of the crosses were advanced to the F_4_ generation without selection. Head-rows in the F_4:6_ generation were bulk harvested and served as seed source for evaluating resistance to WDV in field tests. Populations MI/A40, MU/A40, P1314/A40 and A39/P1314 consisted of 168, 105, 99 and 130 F_4:6_ RILs, respectively.

The winter wheat cultivars Midas, released by Saatzucht Donau GmbH & CoKG (Austria) in 2008, and Mulan, released by NORDSAAT Saatzuchtgesellschaft (Germany) in 2006, and the experimental line P1314 are all highly susceptible to WDV. SVP-72017-17-5-10 (abbreviated to SVP-72017) and P1314 (pedigree: 20812/Hermann) are both semi-dwarf wheat genotypes carrying the *Rht-B1b* allele and have excellent FHB resistance. The breeding line ‘20812’ was selected from the FHB nursery program at IFA Tulln. It descends from a cross with the highly FHB resistant CIMMYT line CM-82036-1TP-10Y-OST-10Y-OM-OFC (abbreviated to CM-82036, pedigree: Sumai-3/Thornbird-S) that is donor of the major FHB resistance QTL *Fhb1* and *Qfhs.ifa-5A* resistance alleles as well as the 1RS.1BL translocation (Buerstmayr et al. 2002, 2003; Samad-Zamini et al. 2017). P1314 has *Fhb1*, *Qfhs.ifa-5A* and the 1RS.1BL translocation*.* Different accessions with the translocation should have an unchanged 1RS chromosome arm, but the 1BL should recombine, apart from any linkage drag in the centromere region.

The experimental line SVP-72017, selected at CPRO-DLO Wageningen, The Netherlands (now Wageningen University and Research) during the 1980s, has the pedigree Marzotto//Dippes Triumph/Mironovskaja 808 and possesses a high level of quantitative resistance to FHB (Buerstmayr et al. 2000; Snijders 1990). SVP-72017 expresses a similar response to WDV as its progenies A39 and A40 (Table 1).

**Table 1:** Best linear unbiased estimator (BLUE), standard deviation (sd), range and line mean heritability coefficient (*H*^*2*^) of wheat dwarf virus response for populations and parental lines

Population	MI/A40	MU/A40	P1314/A40	A39/P1314
Experiment				
2019				
BLUE	6.02	5.11	5.57	5.17
sd	1.31	1.29	1.48	1.53
min	2.65	2.35	2.06	1.66
max	8.57	8.07	8.41	8.09
2020				
BLUE	6.68	5.55	5.81	5.90
sd	1.65	1.59	1.64	1.96
min	2.69	2.59	2.11	1.71
max	9.18	8.29	8.78	9.04
2021				
BLUE	6.58	5.50	6.73	5.68
sd	1.60	1.08	1.43	1.81
min	2.55	2.48	3.22	1.41
max	9.43	7.81	9.16	9.66
Across years				
BLUE	6.42	5.39	6.03	5.59
sd	1.33	1.16	1.4	1.59
min	3.43	3.19	2.49	2.06
max	8.49	7.93	8.39	8.55
*H*^*2*^	0.86	0.86	0.92	0.92

Experiment	2019	2020	2021	Across years
Parents				
SVP-72017	3.23	3.19	5.24	3.89
A39	4.07	3.68	3.00	3.60
A40	5.00	3.88	4.40	4.45
Midas	6.38	6.30	7.67	6.90
Mulan	5.47	5.92	NA^1^	5.77
P1314	6.20	4.10	6.60	6.10

^1^Not available

### Field experiments and disease assessment

RILs of all four populations, including parents and several control lines, were tested in the field at the experimental station of the Department of Agrobiotechnology, Tulln, Austria (latitude 48°18′20ʺN, longitude 16°02′40ʺE, altitude 178 m) in growing seasons 2018/19, 2019/20 and 2020/21. Field trials were arranged as randomized complete block designs with two blocks. Plots consisted of three 1 m rows in 2018/19 and 2019/20 and of six 1 m rows in 2020/21 with 20 cm row spacing. Sowing time was 14th of September in 2018, and 17th of September in 2019 and 2020, approximately four to five weeks earlier compared to usual sowing dates for winter wheat in this region. These early sowing dates resulted in post sowing periods of 50, 41, and 32 days with mean temperatures above 10 °C (Fig. S1), when leaf hoppers are still active allowing for natural infestation of young plants with the virus before winter.

Disease symptoms were visually assessed at heading (BBCH 55) (Lancashire et al. 1991) and at early milk stage (BBCH 73) using a scoring scale from 1 (no or very low disease severity) to 9 (very high severity or dead plants) scale (Table S1). In each year, virus symptomatic leaves of the susceptible control cultivar ‘Capo’ were collected at heading (BBCH 55) and stored at − 80 °C. Leaf samples were analyzed at the JKI Quedlinburg for presence or absence of WDV and barley yellow dwarf virus (BYDV) using sandwich enzyme-linked immunosorbent assay (DAS-ELISA) with polyclonal WDV and BYDV specific antibodies (Clark and Adams 1977).

### Statistical analysis

Statistical analyses were performed in R version 4.0.5 (R Core Team 2020) and were done for each population separately.

#### Field data

Analysis of variance (ANOVA) was conducted for individual years and across years using the R package lme4 (Bates et al. 2015) by applying following linear mixed effects models for single experiments (1) and across years (2).

(1) *P*_*iklmn*_ = *µ* + *g*_*i*_ + *b*_*k*_ + *r*_*l*_*(b)*_*k* +_ *c*_*m*_*(b)*_*k*_ + *ε*_*iklm*_, where *P*_*iklmn*_ denotes the observed phenotypic value, µ the population mean, *g*_*i*_ the effect of genotype *i*, *b*_*k*_ the effect of the block *k*, *r*_*l*_*(b)*_*k*_ the effect of the row *l* nested in the block *k*, *c*_*m*_*(b)*_*k*_ the effect of the column *m* nested in the block *k*, and *ε*_*iklm*_ is the residual.

(2) *P*_*ijklmn*_ = *µ* + *g*_*i*_ + *y*_*j*_ + *gy*_*ij*_ + *b*_*k*_*(y*_*j*_*)* + *r*_*l*_*[by]*_*jk* +_ *c*_*m*_*[by]*_*jk*_ + *ε*_*ijklm*_, where *P*_*ijklmn*_ is the phenotypic observation, µ is the grand population mean, *g*_*i*_ the effect of genotype *i*, *y*_*j*_ the effect of year *j*, *gy*_*ij*_ the interaction between genotype *i* and year *j*, *b*_*k*_*(y*_*j*_*)* the effect of the block *k* nested in the year *j*, *r*_*l*_*[by]*_*jk*_ the effect of the row *l* nested in the block *k* of year *j*, *c*_*m*_*[by]*_*jk*_ the effect of the column *m* nested in the block *k* of year j, *ε*_*ijklm*_ is the residual. The effects of the genotypes were treated as fixed to derive best linear unbiased estimators (BLUEs), while all other effects were modelled as random.

Line mean heritability coefficients were estimated from the variance components of the across year model assuming a random effects model with the equation *H*^2^ = *σ*^2^_*G*_/(*σ*^2^_G_ + *σ*^2^_*GxY*_/*y* + *σ*^2^_*E*_/*yr*), where *σ*^2^_*G*_ is the genotypic variance, *σ*^2^_*GxY*_ the genotype-by-year interaction variance, *σ*^2^_*E*_ the residual variance, *y* the number of years, and *r* the number of replications (Nyquist and Baker 1991). Pearson correlation coefficients between BLUEs of individual years were calculated.

#### Molecular marker analysis and map construction

Genomic DNA was extracted from pooled samples of young leaves (ten plants per RIL and parental line) according to a modified cetyl-trimethyl-ammonium-bromid protocol (Saghai-Maroof et al. 1984). DNA samples were adjusted to a DNA concentration of 50 ng µl^−1^ and genotyping was performed using the 7 K wheat SNP array offered by TraitGenetics GmbH (Gatersleben, Germany, http://www.traitgenetics.com). Marker data and genotypes were quality checked prior to map construction for missing data points, segregation distortion, genotypic duplicates and co-locating markers. RILs sharing more than 95% of markers similarity were combined, markers that showed significant segregation distortion (*p* < 0.001) and more than 20% missing values were excluded from map construction. Genetic maps were calculated using the Minimum Spanning Tree (MST) algorithm (Wu et al. 2008) included in the R package ASMap v0.4 (Taylor and Butler 2017). A *p* value threshold of 1 × 10^−9^ was used to separate markers into linkage groups. Within linkage groups, markers were reordered at a less stringent threshold and recombination frequencies between markers were converted into centiMorgans (cM) using the Kosambi mapping function. To retrieve maximum information on marker data, linkage maps were obtained including co-locating markers, however, for QTL analysis for each co-locating set one marker was chosen as the representative marker and the remaining markers were excluded. Collinearity of linkage groups among the individual populations was checked and markers order were compared with the Wheat IWGSC RefSeq v2.0 (Alaux et al. 2018). Graphical representations of linkage groups and QTL positions were drawn with MapChart 2.2 (Voorrips 2002).

#### QTL analysis

QTL analyses were run on R version 4.0.5 using the package R/qtl 1.50 (Broman et al. 2003). Analyses were done for each year and on BLUEs across years of the averaged WDV scores of both scoring dates. Analyses were conducted for each population separately using the population specific linkage maps. Missing genotypic information was imputed according to the multiple imputation method of Sen and Churchill (2001). Interval mapping was performed to identify main effect QTL and possible epistatic QTL interactions via a genome wide single and two-dimensional QTL scan, respectively, using the Haley–Knott regression method (Haley and Knott 1992). Significance of identified QTL per experiment and population was confirmed by running 1000 permutations for type I error rates at α < 0.1 and α < 0.05. Finally, multiple QTL models (MQM) were fitted including identified QTL. MQM models were explored for the presence of further QTL using addqtl and addint functions. The overall fit of the full model against the null model was tested by ANOVA. The effect of the individual QTL was determined by comparing the full model and the model with the respective term omitted. LOD scores, estimated additive effects and percentage of the phenotypic variance explained by the QTL were obtained from the ANOVA table of the MQM analysis. QTL confidence intervals were determined by a decrease of 1.5-LOD from the position of the LOD peak. QTL identified in individual populations with overlapping intervals were considered identical. Physical Mbp positions of the QTL flanking markers were derived from the IWGSC RefSeq v2.0 (Alaux et al. 2018). RILs were grouped by their QTL combination and the Tukey`s multiple range test was used to compare WDV resistance between groups.

## Results

### Variation in WDV severity

DAS-ELISA tests confirmed infection with WDV and did not identify BYDV. This provided confidence that WDV was the principal pathogen in the field experiments. WDV symptoms were assessed at heading and approximately three weeks later at the early milk stage. Correlation coefficients between WDV severities at the two scoring dates were very high. Coefficients ranged from 0.70 to 0.96 within individual years and from 0.94 to 0.96 for the BLUEs across years (Table S2). We thus combined the information of both WD scoring dates and used the average score as the basis for further analyses and QTL mapping.

**Fig. 1:**
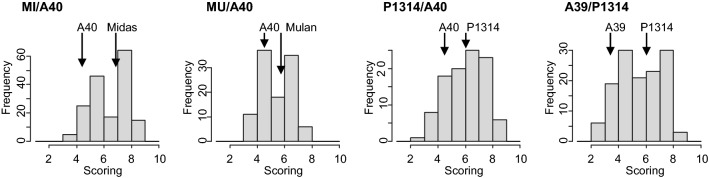
Frequency distributions of wheat dwarf virus (WDV) severity for RIL populations derived from crosses MI/A40, MU/A40, P1314/A40 and A39/P1314. WDV scoring was done on a scale from 1 (no or very weak symptoms) to 9 (severe symptoms or dead plants). Values for the parents are indicated by arrows

All populations showed continuous variation in WDV symptom severity (example image Fig. S2) with a bimodal frequency distribution in populations MI/A40, MU/A40 and A39/P1314 (Fig. 1), suggesting the presence of a major gene. No fully resistant, symptomless wheat line was identified (Table 1). Resistant parents had, on average, a two points lower severity score than susceptible parents on a scale of 1 to 9. Correlation coefficients between years ranged from 0.59 to 0.77, and were lowest in population MU/A40 between years 2019 and 2021 and highest in population A39/P1314 between years 2019 and 2020 (Table S3). Variance components for genotypes were high compared to genotype-by-year interaction (Table S4) resulting in high line mean heritability coefficients with *H*^*2*^ = 0.86 in population MI/A40 and MU/A40 and *H*^*2*^ = 0.92 in populations P1314/A40 and A39/P1314 (Table 1).

### Linkage maps

After quality filtering, 2006, 2466, 2423 and 2216 markers remained for map construction of population MI/A40, MU/A40, P1314/A40 and A39/P1314, respectively. The resulting genetic linkage maps comprised 34, 45, 47 and 40 linkage groups of total lengths 2935, 2838, 2881 and 3100 cM representing all 21 wheat chromosomes (Tables S5, S6). Three hundred and eighty-four markers were polymorphic in all four populations; additionally, 1063 and 1381 polymorphic markers were shared between three and two populations, respectively, whereas 1624 markers showed population specific polymorphism (Fig. S3; Table S5). Distributions of recombination events yielded 574, 619, 629 and 659 recombination points (loci) with average bin sizes of 5.1, 4.6, 4.6 and 4.7 cM within maps MI/A40, MU/A40, P1314/A40 and A39/P1314, respectively (Table S6). Comparison of marker alignments with the IWGSC RefSeq v2.0 showed high agreement between physical and genetic maps.

### QTL analysis

**Fig. 2:**
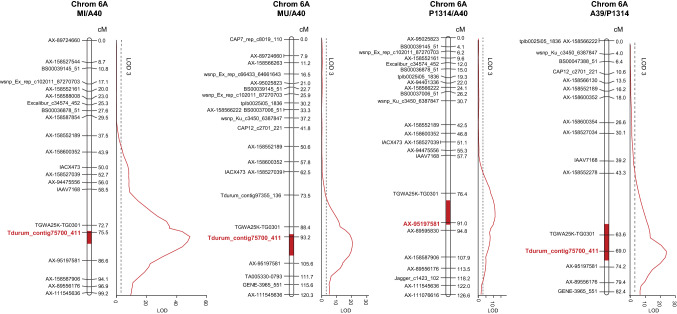
Genetic maps of chromosome 6A for populations MI/A40, MU/A40, P1314/A40 and A39/P1314. LOD profiles of BLUEs across experiments for resistance to wheat dwarf virus (WDV) were determined by the MQM model. Only subsets of markers are shown. Markers closest to the QTL peaks are highlighted in red and bold. Sizes of the QTL support intervals are indicted by red bars within chromosomes and refer to a LOD decrease of 1.5 from maximum LOD

QTL analysis identified two QTL: *Qwdv.ifa-6A* on the long arm of chromosomes 6A and *Qwdv.ifa-1B* on chromosome 1B. *Qwdv.ifa-6A* had a strong effect in all experiments of all populations, and explained on average 45.5, 59.8, 64.4 and 73.9% of the phenotypic variance in the individual mapping populations (Table 2). The QTL mapped in all populations to the same region that covered a genetic distance of 5, 10, 12 and 6 cM in population MI/A40, MU/A40, P1314/A40 and A39/P1314, respectively (Fig. 2). Taking the information for all four maps and the physical positions of markers in IWGSC RefSeq v2.0 the QTL became a ~ 6.5 Mbp interval limited by markers TGWA25K-TG0301 (599,366,272 bp) and AX-95197581 (605,868,853 bp) with Tdurum_contig75700_411 (601,412,152 bp) as the marker closest to the QTL peak.

**Table 2:** Location and estimates of QTL for BLUEs of wheat dwarf virus (WDV) resistance in single years and across years using multiple QTL mapping

Population Experiment	QTL	Peak	interval	Flanking markers							
	Chrom	cM	cM	Upper	Mbp^a^	Lower	Mbp^a^	LOD^b^	PV%^c^	add^d^	sign^e^
MIxA40											
Across years	6A	76	75–80	TGWA25K-TG0301	599.37	AX-95197581	605.87	48.9	73.9	1.12	***
2019	6A	76	74–80	TGWA25K-TG0301	599.37	AX-95197581	605.87	28.1	53.8	0.95	***
2020	6A	76	74–80	TGWA25K-TG0301	599.37	AX-95197581	605.87	36.6	63.3	1.30	***
2021	6A	76	73–80	TGWA25K-TG0301	599.37	AX-95197581	605.87	23.5	47.5	1.11	***
MUxA40											
Across years	6A	96	92–102	Tdurum_contig75700_411	601.41	AX-95197581	605.87	20.6	59.8	0.91	***
2019	6A	98	90–104	Tdurum_contig75700_411	601.41	AX-95197581	605.87	12.9	43.4	0.88	***
2020	6A	96	90–102	Tdurum_contig75700_411	601.41	AX-95197581	605.87	16.7	52.3	1.18	***
2021	6A	93	92–100	Tdurum_contig75700_411	601.41	AX-95197581	605.87	12.0	41.3	0.71	***
P1314xA40											
Across years	6A	88	80–92	TGWA25K-TG0301	599.37	AX-89595830	607.34	15.4	45.5	0.96	***
2019	6A	84	76–94	TGWA25K-TG0301	599.37	AX-89595830	607.34	12.4	40.4	0.96	***
2020	6A	88	78–94	TGWA25K-TG0301	599.37	AX-89595830	607.34	11.1	34.6	1.02	***
2021	6A	88	82–94	TGWA25K-TG0301	599.37	AX-89595830	607.34	10.2	35.0	0.86	***
P1314xA39											
Across years	6A	68	66–72	TGWA25K-TG0301	599.37	AX-95197581	605.87	30.8	64.4	1.27	***
2019	6A	68	66–72	TGWA25K-TG0301	599.37	AX-95197581	605.87	24.0	58.9	1.48	***
2020	6A	68	66–72	TGWA25K-TG0301	599.37	AX-95197581	605.87	21.2	54.0	1.35	***
2021	6A	69	66–72	TGWA25K-TG0301	599.37	AX-95197581	605.87	17.5	43.6	1.01	***
P1314xA40											
Across years	1B	0	0–10	BS00050522_51	1.4217	GENE-3626_308	458.69	4.99	11.3	− 0.47	***
2019	1B	0	0–14	BS00050522_51	1.4217	CAP12_c1129_220	542.41	2.85	7.3	− 0.41	***
2020	1B	0	0–8	BS00050522_51	1.4217	GENE-3626_308	458.69	5.59	15.1	− 0.65	***
P1314xA39											
Across years	1B	0	1–4	TGWA25K-TG0025	151.76	Tdurum_contig70304_781	453.29	11.7	15.8	− 0.61	***
2019	1B	0	1–8	TGWA25K-TG0025	151.76	Tdurum_contig70304_781	453.29	8.96	18.5	− 0.67	***
2020	1B	0	1–4	TGWA25K-TG0025	151.76	Tdurum_contig70304_781	453.29	5.91	9.73	− 0.57	***
2021	1B	0	1–4	TGWA25K-TG0025	151.76	Tdurum_contig70304_781	453.29	5.35	9.65	− 0.57	***

^a^Mega base pair position according IWGSC RefSeq V2.0

^b^LOD (logarithm of the odds)

^c^Percentage of phenotypic variance explained by the QTL

^d^Positive additive effect denote resistance improving effect by the A40 or A39 alleles; additive effects were estimated as half the difference between phenotype averages for the homozygote

^e^ *** significant at *p* < 0.001

Haplotype comparison revealed a unique common haplotype for A40, A39 and SVP-72017 across the 6A QTL interval (Table S7), suggesting that WDV resistance originated from the SVP-72017 ancestral line.

**Fig. 3:**
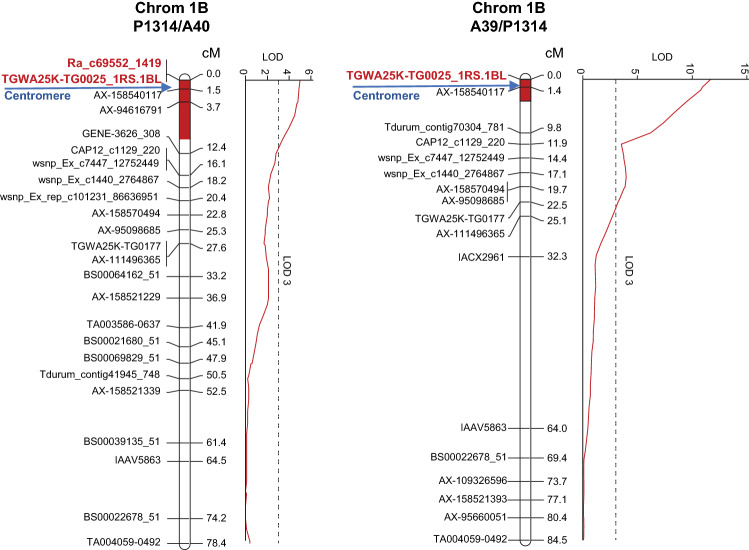
Genetic maps of chromosome 1B for population P1314/A40 and A39/P1314. LOD profiles of BLUEs across experiments for resistance to wheat dwarf virus (WDV) were determined by the MQM model. Only subsets of markers are shown. Markers closest to the QTL peaks are highlighted in red and bold. Sizes of the QTL support intervals are indicted by red bars within chromosomes and refer to a LOD decrease of 1.5 from maximum LOD. Approximate positions of the centromeres are indicated by arrows

The second QTL mapped to chromosome 1B. The resistance-improving allele derived from the susceptible parent P1314; hence, the QTL was identified only in population P1314/A40 and A39/P1314. It was identified in all years in population A39/P1314, explaining between 9.6 and 18.5% of the phenotypic variance (Table 2). *Qwdv.ifa-1B* was less constant in population P1314/A40; it was detected in year 2019, 2020 and in the mean across all experiments and contributed between 7.3 and 15.1% to the phenotypic variance. *Qwdv.ifa-1B* covered a genetic distance of 10 cM in population P1314/A40 and 4 cM in A39/P1314 (Fig. 3; Table 2). ‘P1314’ and ‘20812’ had the same haplotype across the *Qwdv.ifa-1B* support interval encompassing the entire short arm and the pericentromeric region of the long arm up to AX-94433968 at bp position 339,560,059, suggesting that the WDV resistance originated from experimental line 20812. The highly FHB resistant CIMMYT line CM-82036 (parental line of 20812) and 20812 share the same haplotype on 1BS until marker AX-110366212 (bp position 295,069,625) (Table S8). CM-82036 contains the 1RS.1BL translocation (Samad-Zamini et al. 2017). The SNP marker TGWA25K-TG0025 was developed by TraitGenetics GmbH and detects the 1RS.1BL translocation (personal communication). P1314 and 20812 as well as CM-82036 share the same SNP haplotype for the TGWA25K-TG0025 marker, confirming the presence of the rye chromatin in these lines. *Qwdv.ifa-1B* may therefore be associated with the wheat/rye translocation.

**Fig. 4:**
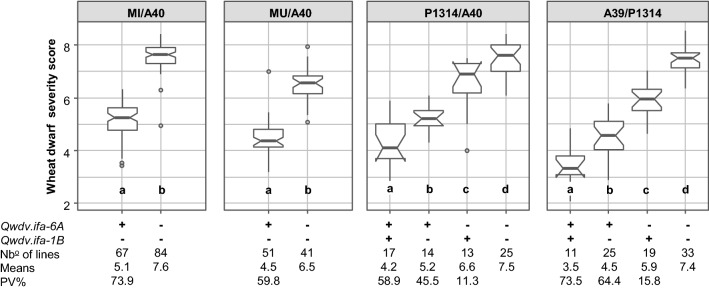
Box plot distributions of wheat dwarf virus severities for RILs of populations MI/A40, MU/A40, P1314/A40 and A39/P1314 grouped by their QTL combinations. Medians are indicated by *solid bold lines*, *open circles* represent outliers. For each population, QTL combination, the number of lines, mean values and phenotypic variance (PV%) explained by the QTL or QTL combination are provided. QTL groups with different letters are significantly different (*p* < 0.05) based on Tukey HSD test. Boxplots are based on BLUEs of means across years. WDV scoring scale ranged from 1 (no or very weak symptoms) to 9 (severe symptoms or dead plants)

*Qwdv.ifa-6A* and *Qwdv.ifa-1B* acted additively, no epistatic interactions were observed (Fig. 4). Both QTL combined explained 58.9 and 73.5% of the phenotypic variance for the mean across experiments in populations P1314/A40 and A39/P1314, respectively. Comparing RILs grouped by their QTL status showed significant and strong differences between QTL groups in all populations. RILs having the QTL combined were most resistant, followed by lines carrying the *Qwdv.ifa-6A* resistance. *Qwdv.ifa-6A* was significantly more effective than *Qwdv.ifa-1B*, and RILs with no resistance QTL were most diseased. The effect of *Qwdv.ifa-6A* was particularly strong in the A40/MI and A40/MU populations, where *Qwdv.ifa-6A* was the only detected QTL.

## Discussion

### Using early autumn sowing for natural WDV infection revealed highly reproducible phenotypic data

In our study, natural WDV infestation was provoked by sowing four to five weeks earlier than routinely practiced in the test region. This mimicked unusually long periods of warm autumn temperature that are predicted to occur more frequently in the future due to climate change. Dates of sowing resulted in five to seven-week periods post sowing with mean temperatures above 10 °C. This facilitated seed germination and first leaf development when the leafhopper vector were still active. In this scenario leaf hoppers that have already acquired WDV from infected plants during the summer season can infect young plants when they are most susceptible making early sowing a simple and efficient alternative to artificial WDV inoculation. Sowing in early autumn has indeed proven to be very reliable, since infection levels were high and uniform across experiments, evidenced by high correlation coefficients between years (Table S3), low variance components for the year-by-genotype interactions (Table S4) and high heritability coefficients (Table 1). Examining symptomatic plants for presence of WDV and BYDV using ELISA tests revealed that WDV was the causal pathogen in each season.

There is a great lack of knowledge regarding sources and genetic basis of WDV resistance in wheat. No completely resistant genotype has been identified so far, most wheat cultivars are highly susceptible and only a few cultivars have moderate to high partial resistance to WDV (Benkovics et al. 2010; Nygren et al. 2015; Pfrieme et al. 2022). Similarly, we observed continuous variation in WDV severity, but could not identify a fully resistant genotype among parents and the more than 500 RILs tested (Fig. 1; Table 1).

### QTL analysis identifies two major WDV resistance QTL

Resistance to WDV was controlled by the highly effective QTL *Qwdv.ifa-6A* and the moderately to highly effective QTL *Qwdv.ifa-1B* (Figs. 2, 3, 4; Table 2). *Qwdv.ifa-6A* had a strong effect in all experiments and was mapped to chromosome 6AL to an interval of approximately 6.5 Mbp with Tdurum_contig75700_411 (601.4 Mbp) as the peak marker. The favorable allele traced back to the experimental line SVP-72017 (Table S7). GWAS revealed a QTL for BYDV resistance in a similar region, with the closest marker, IWB69770, 4.2 Mbp proximal to the peak marker identified in our study (Choudhury et al. 2019). Whether this coincidence is due to two closely linked QTL or to a single QTL that has a pleiotropic effect on several viral pathogens remains unclear.

The second QTL *Qwdv.ifa-1B* mapped to chromosome 1B and its resistance derived from the susceptible parent P1314. Because of the 1RS.1BL translocation in P1314 no recombinants occurred on the short arm, resulting in a large discrepancy between genetic and physical distances. The relatively short QTL support interval of 4 to 10 cM involved the entire short arm and part of the pericentromeric region of the long arm (Fig. 3; Table 2). The *Qwdv.ifa-1B* support interval overlaps with the 1RS.1BL translocation suggesting that WDV resistance is most likely conferred by the rye chromatin of this translocation. The 1RS.1BL chromosome has been widely used in wheat breeding for its potential to improve adaptability, stability, yield and disease resistance (Rabinovich 1998; Villareal et al. 2006). Many disease resistance genes, e.g. genes for resistance to leaf rust (*Lr26*), stripe rust (*Yr9*), stem rust (*Sr31*) and powdery mildew (*Pm8*) were transferred into wheat through the 1RS.1BL translocation. Unfortunately, since the widespread use of this translocation, virulent isolates of the powdery mildew and rust pathogens have evolved. The 1RS.1BL rye chromatin segment was reported to be effective against wheat streak mosaic virus; its presence caused delayed symptom development and reduced spread of the virus (Li et al. 2007); however, no evidence that the same gene causes resistance to WDV and wheat streak mosaic virus has been reported yet.

The GWAS study conducted by Pfrieme et al. (2022) identified 35 significant marker trait associations on 10 different chromosomes, suggesting a polygenic regulation of WDV resistance. For validation of the identified associations, four populations were generated using the Russian wheat cultivar ‘Fisht’ as resistant parent and 14 significant effects were confirmed. Interestingly, five of the significant QTL mapped to the short arm of chromosome 1B and thus overlapped with *Qwdv.ifa-1B* identified in our study. Fisht has the favorable allele on chromosome 1B, but there is no information regarding presence or absence of the 1RS.1BL translocation. It is therefore unclear, whether Fisht and P1314 (resistance donor for *Qwdv.ifa-1B*) share the same resistance gene. There is a clear additivity of *Qwdv.ifa-6A* and *Qwdv.ifa-1B* (Fig. 4). This suggests that pyramiding resistance QTL will increase both durability and degree of resistance. The ancestral grandparent lines SVP-72017 and 20812 are both highly resistant to FHB. Currently, the populations are being tested for FHB resistance with the aim to identify RILs that are highly resistant to both WDV and FHB. Such RILs would be of great value as parents for WDV and FHB resistance breeding.

## Conclusion

Relying on late sowing for controlling WDV will not be effective if long periods of warm temperatures persist during late autumn and early winter. Moreover, late sowing causes serious problems in organic farming, where the bunts are a significant problem. Resistant cultivars would be the preferred management strategy, but most current wheat cultivars are susceptible or highly susceptible to WDV. Therefore, improving resistance to WDV in winter wheat is of great importance. We report here two effective resistance QTL that can be integrated in breeding programs by means of marker-assisted selection. In addition, we demonstrate that provocation of natural infection by early sowing provides a uniform infection pressure allowing reproducible and reliable WDV phenotyping. Thus, it is a simple and efficient alternative to tedious and time-consuming artificial infection with WDV-bearing leafhoppers or nymphs and is readily applicable in breeding programs.

## Supplementary Information

Below is the link to the electronic supplementary material.

Supplementary file1 (PDF 885 KB)

Supplementary file2 (PDF 114 KB)

Supplementary file3 (XLSX 344 KB)

## Data Availability

The plant material and datasets employed in this study are available from the corresponding author on reasonable request.
