# Supplementary material for: Two major quantitative trait loci control wheat dwarf virus resistance in four related winter wheat populations
Source: Theor Appl Genet. 2023 Apr 7;136(5):103. doi: 10.1007/s00122-023-04349-3 (PMC10082126; doi:10.1007/s00122-023-04349-3)
Supplement: Supplementary file 1 — Supplementary file1 (PDF 885 KB) [file 122_2023_4349_MOESM1_ESM.pdf]

Online Resource 1

Article title: Two major quantitative trait loci control wheat dwarf virus resistance in four related winter wheat populations

Journal: Theoretical and applied genetics

Authors: Maria Buerstmayr, Hermann Buerstmayr

Name, affiliation, and email of corresponding author:

Hermann Buerstmayr, University of Natural Resources and Life Sciences, Vienna, Institute of Biotechnology in Plant Production, Konrad Lorenz Straße 20, 3430 Tulln, Austria
e-mail: hermann.buerstmayr@bouk.ac.at

Figure S1 Mean temperature (°C) at IFA Tulln between 1st of September and 30th of November for the years 2018, 2019 and 2020

Figure S2 Example image showing the variation in the expression of wheat dwarf severity among winter wheat lines

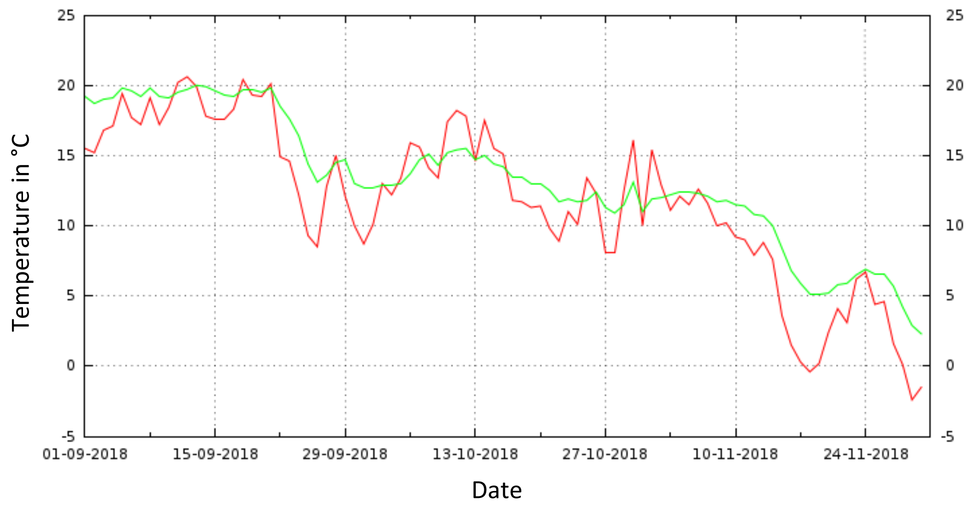

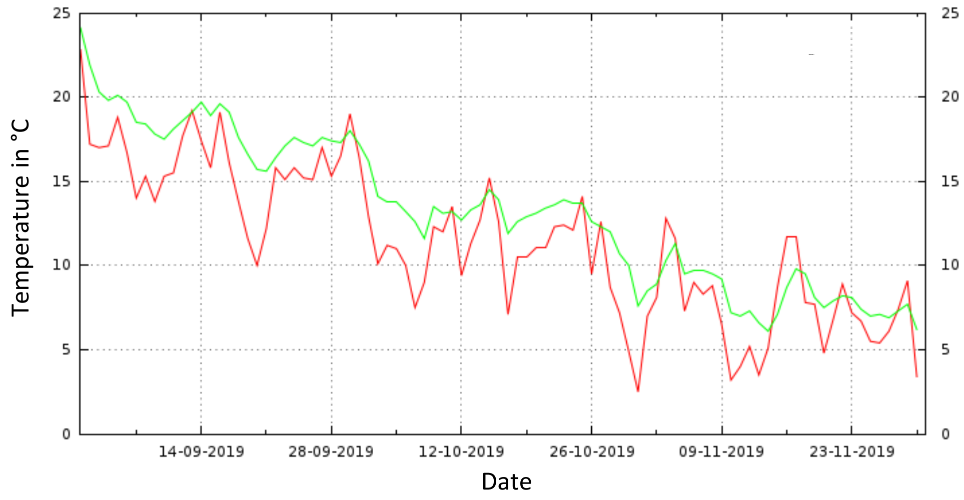

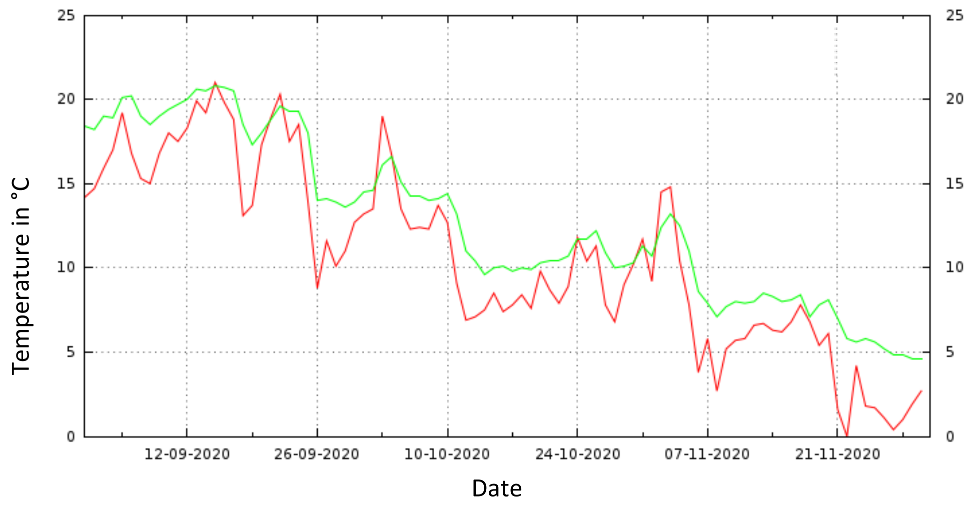

Figure S1 Mean temperature (°C) 2 m above soil surface (red solid line) and mean soil temperature (green solid line) at IFA Tulln between 1st of September and 30th of November for the years 2018, 2019 and 2020

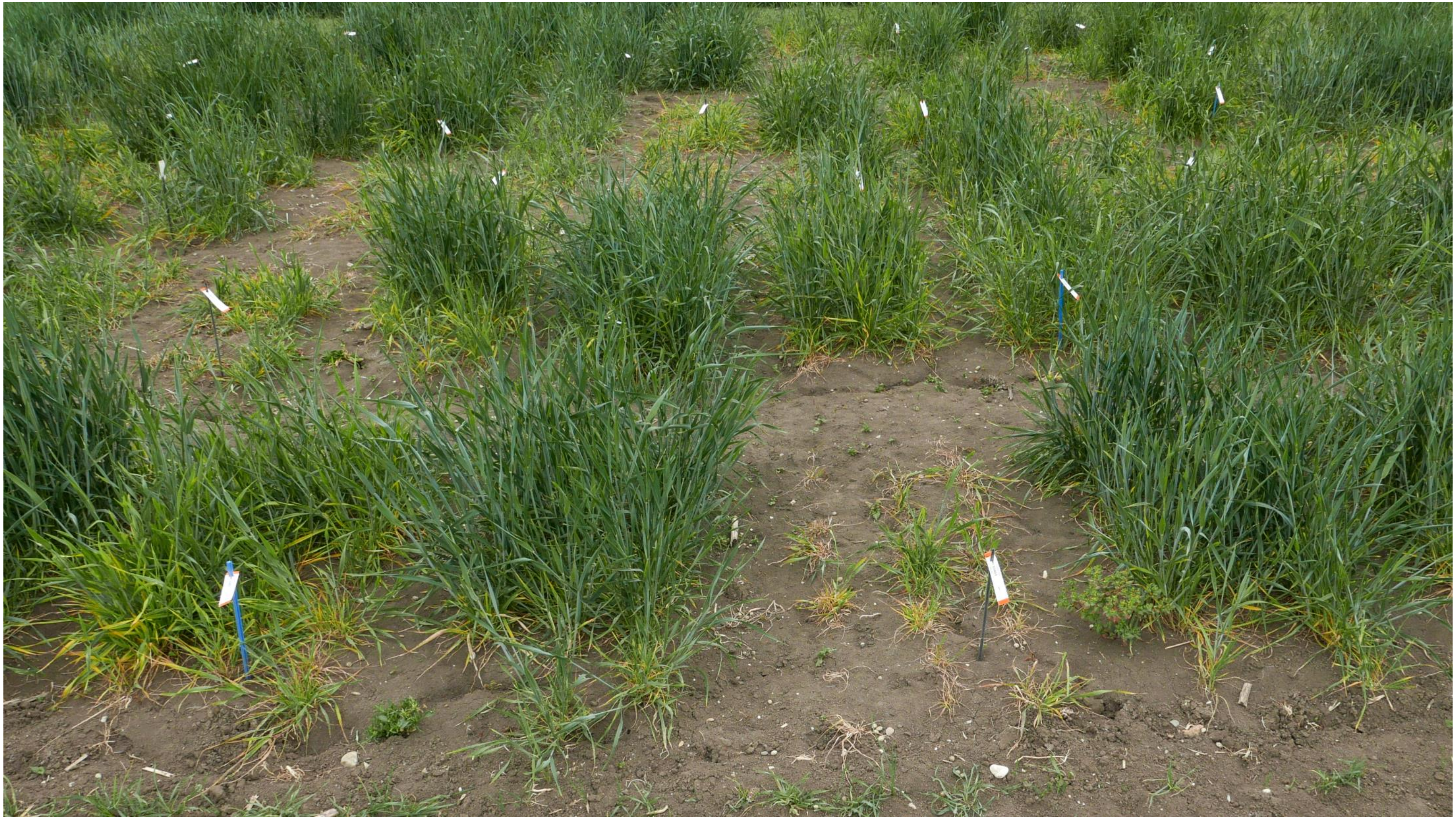

Figure S2 Example image showing the variation in the expression of wheat dwarf severity among winter wheat lines
