# Supplementary material for: Two major quantitative trait loci control wheat dwarf virus resistance in four related winter wheat populations
Source: Theor Appl Genet. 2023 Apr 7;136(5):103. doi: 10.1007/s00122-023-04349-3 (PMC10082126; doi:10.1007/s00122-023-04349-3)
Supplement: Supplementary file 2 — Supplementary file2 (PDF 114 KB) [file 122_2023_4349_MOESM2_ESM.pdf]

Online Resource 2

Article title: Two major quantitative trait loci control wheat dwarf virus resistance in four related winter wheat populations

Journal: Theoretical and applied genetics

Authors: Maria Buerstmayr, Hermann Buerstmayr

Name, affiliation, and email of corresponding author:

Hermann Buerstmayr, University of Natural Resources and Life Sciences, Vienna, Institute of Biotechnology in Plant Production, Konrad Lorenz Straße 20, 3430 Tulln, Austria
e-mail: hermann.buerstmayr@boku.ac.at

Content: Table S1 – Table S4

Table S1 Applied wheat dwarf virus (WDV) severity scoring scheme

Table S2 Correlation coefficients between BLUEs for wheat dwarf virus severity of the first and the second scoring date

Table S3 Correlation coefficients of wheat dwarf virus severity between years

Table S4 Variance component estimates for wheat dwarf virus severity across three years for populations MI/A40, MU/A40, P1314/A40 and A39/P1314

Table S1 Applied wheat dwarf virus (WDV) severity scoring scheme

Score	Severity in % clearly WDV diseased plants
1	< 10 %
2	10-20%
3	20-30%
4	30-40%
5	40-50%
6	50-60%
7	60-70%
8	70-80%
9	> 80%

Table S2 Correlation coefficients between BLUEs for wheat dwarf virus severity on the first and the second scoring date

	Population			
Year	MI/A40	MU/A40	P1314/A40	A39/P1314
2019	0.89	0.89	0.91	0.86
2020	0.96	0.93	0.94	0.94
2021	0.88	0.70	0.86	0.84
Across years	0.96	0.94	0.96	0.96

Table S3 Correlation coefficients of wheat dwarf virus severity between years. For comparison, the BLUEs of the average values for both assessment dates were used

Year comparisons	MI/A40	MU/A40	P1314/A40	A39/P1314
2019 : 2020	0.71	0.75	0.75	0.77
2019 : 2021	0.65	0.59	0.76	0.73
2020 : 2021	0.63	0.61	0.71	0.74

Table S4 Variance component estimates for wheat dwarf symptom severity across three years for populations MI/A40, MU/A40, P1314/A40 and A39/P1314

	Population			
Variance component	MI/A40	MU/A40	P1314/A40	A39/P1314
σ^2 Genotype	1.50***	1.13***	1.75***	2.29***
σ^2 Year	0.00	0.00	0.00	0.00
σ^2 Genotype*Year	0.22	0.09	0.38	0.09
σ^2 Block within Year	0.53	0.96	0.15	0.28
σ^2 Column within Block within Year	0.11	0.24	0.29	0.15
σ^2 Row within Block within Year	0.21	0.25	0.13	0.19
σ^2 Error	1.03	0.89	0.96	1.08

*** $p < 0.001$
